# Treatment Satisfaction with Nonoperative Management of Suspected Hip Fractures in Nursing Home Patients with a Do-Not-Hospitalize Directive: A Prospective Case Series (NONU-HIP)

**DOI:** 10.1177/08258597251375240

**Published:** 2025-09-24

**Authors:** Sverre A.I. Loggers, Romke Van Balen, Jeroen Steens, Hanna C. Willems, Pamela Riezebos, Anja Wagenaar-Huisman, Michael H.J. Verhofstad, Esther M.M. Van Lieshout, Pieter Joosse

**Affiliations:** 1 Department of Surgery, 1140 Northwest Clinics Alkmaar, AM Alkmaar, the Netherlands; 2 Trauma Research Unit Department of Surgery, Erasmus MC, University Medical Center Rotterdam, CA Rotterdam, the Netherlands; 3 Department of Public Health and Primary Care, Leiden University Medical Center, RC Leiden, the Netherlands; 4 Department of Orthopaedic Surgery, 3956 Dijklander Ziekenhuis, AR Hoorn, the Netherlands; 5 Geriatrics Section, Department of Internal Medicine, Amsterdam UMC location AMC, Amsterdam, the Netherlands; 6 621281 Medical department Omring, Hoorn, the Netherlands; 7 Medical department Wilgaerden, Wognum, the Netherlands; 8 Department of Surgery, Rode Kruis Ziekenhuis, Beverwijk, the Netherlands

**Keywords:** Quality of dying, proximal femoral fracture, palliative care, nursing home, hip fracture, nonoperative

## Abstract

**Objectives:**

Some nursing home residents opt to forgo hospital admission in case of a suspected hip fracture due to the poor prognosis. However, outcomes in these patients are unknown and hamper advance care planning and expectation management. This study assesses treatment satisfaction and quality of life in nursing home residents with a suspected hip fracture and a do-not-hospitalize directive.

**Methods:**

A prospective case series study was conducted in three nursing home organizations in The Netherlands. The primary outcome was the treatment satisfaction according to proxies and caregivers. Secondary outcomes were EuroQoL-5D-5L utility score, Qualidem scores, pain and opiate administration, adverse events, mortality, and quality of dying (Quality of Dying and Death Questionnaire).

**Results:**

Twenty patients, with a median age of 87 years, were included. The treatment satisfaction as rated by proxies and caregivers was high (median numeric rating scale of 9 [P25-P75 8-10] and 9 [P25-P75 8-9], respectively). The life expectancy was short (median of 5 days [P25-P75 3-6]) with a 14-day mortality rate of 100%. The overall quality of the death was “good to almost perfect” in 77% of the patients (n = 10/13) and “intermediate” in 23% (n = 3/13). Symptom control was rated as “good to almost perfect” in 70% of patients. Four adverse events occurred in three patients (15%).

**Conclusions:**

This study showed that nonoperative management of suspected proximal femoral fractures in nursing home patients that opted to forgo hospital admission, results in high treatment satisfaction, high quality of dying with good symptom control, and predictable short-term mortality rates.

## Introduction

Proximal femoral fractures in nursing home patients are associated with poor outcomes with regard to regaining pre-fracture functioning and mortality.^1^ Hospital admission, and mainly surgery, in this group often results in cognitive decline, delirium, and adverse events.^1,2^ In some nursing home patients, especially those with advanced dementia and a limited life expectancy, a palliative-centered approach may become more appropriate than curative care.^3–6^

In these patients with a suspected hip fracture in the final phase of life, the decision can be made for nonoperative management (NOM) with a do-not-hospitalize (DNH) directive via advance care planning (ACP) or in consultation with the patient or legal representatives at the time of a suspected fracture. Consequently, these patients will not be referred to a hospital for diagnostics or treatment. The current rate of non-hospitalization of nursing home residents with a suspected proximal femoral fracture is unknown. However, a survey study among a general population of nursing home residents in the Netherlands showed that, when ACP with proper counseling was done, 50% of the residents or their legal representatives would opt for NOM without referral to the hospital for admission, diagnostic tests, and/or interventions.^7^

Hypothetically, NOM without hospitalization in nursing home residents with a suspected proximal femoral fracture would spare the patients from unnecessary transfers, diagnostic procedures, risks associated with hospital admission, and ultimately reduce healthcare costs without altering the quality of care while patients can remain in their own familiar environment. This would mean supportive care with adequate analgesia with goals of care focused on comfort and quality of life (QoL) in the final days or weeks of their lives. However, the outcome of NOM of suspected proximal femoral fractures with regard to treatment satisfaction of caregivers and relatives, the QoL, and life expectancy are unknown for those who are not admitted to a hospital and treated in their own nursing home environment. This leads to practice variation and unwanted differences in education and expectation management of the patients and relatives.

The aim of this study was to investigate the treatment satisfaction and (health-related) quality of life ((HR)QoL) in nursing home residents with a suspected proximal femoral fracture with a DNH directive. This will aid decision making for potential hospitalization, ACP, and for realistic expectation management after a suspected fracture.

## Methods

A prospective case series study was conducted in three nursing home organizations in The Netherlands between November 1, 2019 and December 31, 2022. All permanently institutionalized nursing home residents aged over 65 years with a suspected proximal femoral fracture as judged by the elderly care physicians (eg, shortening and/or external rotation of the affected leg, inability to mobilize, and pain on manipulation of the hip) who underwent NOM while forgoing hospital admission were included. Patients were excluded if provision of informed consent by the patient or proxies was not obtained within 4 days of the day of trauma. Also patients that participated in another surgical intervention or drug study that might influence any of the outcome parameters were excluded. Patients were planned to be followed until a maximum of 3 months post-injury with scheduled visits on day 3, day 7, day 14, after 1 month, and 3 months. The prospective design and points of measurement was chosen in order to gather real-time data during the initial and most important period after the fracture and minimize loss of data which is based upon the short life expectancy in a previous study.^2^

The study was registered at the Netherlands Trial Register (NL8012: date 02-09-2019). The Medical Research Ethics Committee of VUmc exempted the study (2019.343). Patients or proxies provided written consent for participation.

Treatment decisions for NOM were either made via DNH directives from ACP or with patients or legal representatives at the time of sustaining the injury by the treating elderly care physicians or nurse practitioner. After the decision was made to not hospitalize the frail nursing home resident with a short life expectancy and with the suspected proximal femoral fracture, informed consent was obtained to participate in this follow-up study.

### Outcome Measurements

The primary outcome measure was the treatment satisfaction with the chosen NOM strategy with DNH directive according to the proxy (relatives or legal representative) and caregiver (treating elderly care physicians or nurse practitioner). Treatment satisfaction was assessed on a 0 to 10 numeric rating scale (NRS). Herein, 0 was extremely dissatisfied and 10 extremely satisfied.

The secondary outcome measures were ((HR)QoL), degree of neuropsychiatric symptomatology, the level of pain and analgesic drug use, time to death, adverse events, mobility, and the quality of dying and death (QODD).

HRQoL was assessed with the EuroQoL-5D-5L (EQ-5D) utility score. The EQ-5D is often used for femoral fracture research and can be completed by both patients and proxies with good proxy-patient agreement.^8–11^ Questionnaires completed by proxies were inevitable because of the degree of cognitive impairment of the intended study population. The patient's EQ-5D-5L was converted into utility scores using the Dutch tariff, with lower scores indicating poorer HRQoL ranging from −0.11 to 1.^12^ The EQ-5D was assessed at day 3, 7, and 14.

The Qualidem was used to measure the QoL on nine domains in persons with dementia. It is a 37-item questionnaire completed by caregivers covering the last 7 days. The items are rated on a four-point Likert scale.^13^ Higher scores indicate better QoL, with a minimum score of 0 and maximum scores between 6 and 21 per domain. Qualidem scores are mainly useful in order to compare groups or interventions in patients with dementia and less useful in non-comparative studies but this questionnaire was included to allow comparison in future studies.

The degree of neuropsychiatric symptoms (ie, anxiety, restlessness, etc.) were assessed by a Dutch translation of the Neuropsychiatric Inventory–Questionnaire (NPI-Q).^14^ The NPI-Q was developed and validated to provide a brief assessment of neuropsychiatric symptomatology in routine clinical practice settings. Each of the 12 NPI-Q domains contains a survey question in which initial responses to each domain question are present or absent. If present, the informant then rates both the Severity of the symptoms present within the last month on a 3-point scale and the associated impact of the symptom manifestations on them (ie, Caregiver Distress) using a 5-point scale.

Pain was assessed using the Pain Assessment Checklist for Seniors with Limited Ability to Communicate (PACSLAC), which is a proven useful 24-item instrument for recognizing pain in patients with dementia.^15^ The total score ranges from 0 to 24, resulting in a dichotomous outcome with a score of four points or higher indicating pain. This was assessed in rest and during nursing activities. In addition, the daily amount of narcotic drug administration was calculated using the equivalence scale for 1 mg oral morphine per day. Adverse events during the follow-up period were registered with corresponding severity according to the Clavien-Dindo classification.^16^

If a patient had died, their proxies were asked to participate in an interview about the QODD. This interview-based questionnaire explores 17 end-of-life priorities resulting in three corresponding categories of the quality of the final period of the decedent's life (“terrible to poor,” “intermediate,” and “good to almost perfect”).^17,18^

Pre-injury patient characteristics were established via patient chart reviewing and interviews with the treating elderly care physicians or nurse practitioners. Additional patient characteristics were: age, gender, Charlson Comorbidity Index, Activities of daily living (ADL) dependency measure by the KATZ-ADL score, pre-fracture mobility, and cognitive function. The pre-trauma cognitive function of participants was established at the day 3 visit with the Clinical Dementia Rating scale and Cognitive Performance Scale.^19,20^

### Statistical Analysis

Data were analyzed using the Statistical Package for the Social Sciences (SPSS) version 25.0 (SPSS, Chicago, Ill., USA) and reported following the Strengthening the Reporting of Observational studies in Epidemiology guidelines. Only descriptive analyses were performed. Continuous data were reported as median and inter quartile ranges (due to non-normal distribution), categorical data as number with percentages.

## Results

Twenty patients were included during the study period with a median age of 87 years (Table 1). Half of the patients (n = 10) had moderate-severe dementia while all patients had some degree of ADL dependency pre-trauma (Table 1). In none of the cases, a consultation with an in-hospital consultant was conducted to discuss potential hospitalization. A predefined DNH directive was in place in nine patients (45%), and in the other 11 cases (55%), this was decided on after shared decision making with the patient and/or family upon the clinical suspicion of the hip fracture.

**Table 1.** Patient Characteristics.

Characteristic	N	Total group (N = 20)
Age (years)	20	87 [83-92]
Sex (female)	20	16 (80%)
BMI (kg/m2)	13	20,9 [19.9-22,1]
BMI <18.5 kg/m^2^	13	5 (39%)
CCI	20	5 [2-6]
ASA-score ASA II	20	2 (10%)
ASA III		15 (75%)
ASA IV		3 (15%)
CDR scale absent	19	2 (11)
Present, but mild		7 (37%)
Moderate		5 (26%)
Severe		5 (26%)
CPS scale	18	3 [2-5]
Mobility FAC ≤2	20	5 (25%)
Mobility bed-bound	20	0 (%)
Bed-chair transfer		2 (10%)
Few steps (AR < 10 m)		2 (10%)
Mobile (AR ≥10 m)		16 (80%)
KATZ-ADL 0 (ADL independent)	20	0 (0%)
1		2 (10%)
2		1 (5%)
3		2 (10%)
4		7 (35%)
5		5 (25%)
6 (Total dependency)		3 (15%)

Data are presented as median [P_25_-P_75_] or as n (%).

BMI, body mass index; CCI, Charlson comorbidity index; ASA-score, American Society of Anesthesiologists Score; CDR scale, clinical dementia rating; CPS scale, cognitive performance scale; FAC, functional ambulatory category; AR, action radius; KATZ-ADL, KATZ activities of daily living.

The treatment satisfaction as rated by proxies and caregivers was high. Proxies rated the treatment satisfaction with a median NRS of 9 [8-10] and caregivers with a median NRS of 9 [8-9].

**Figure 1.**
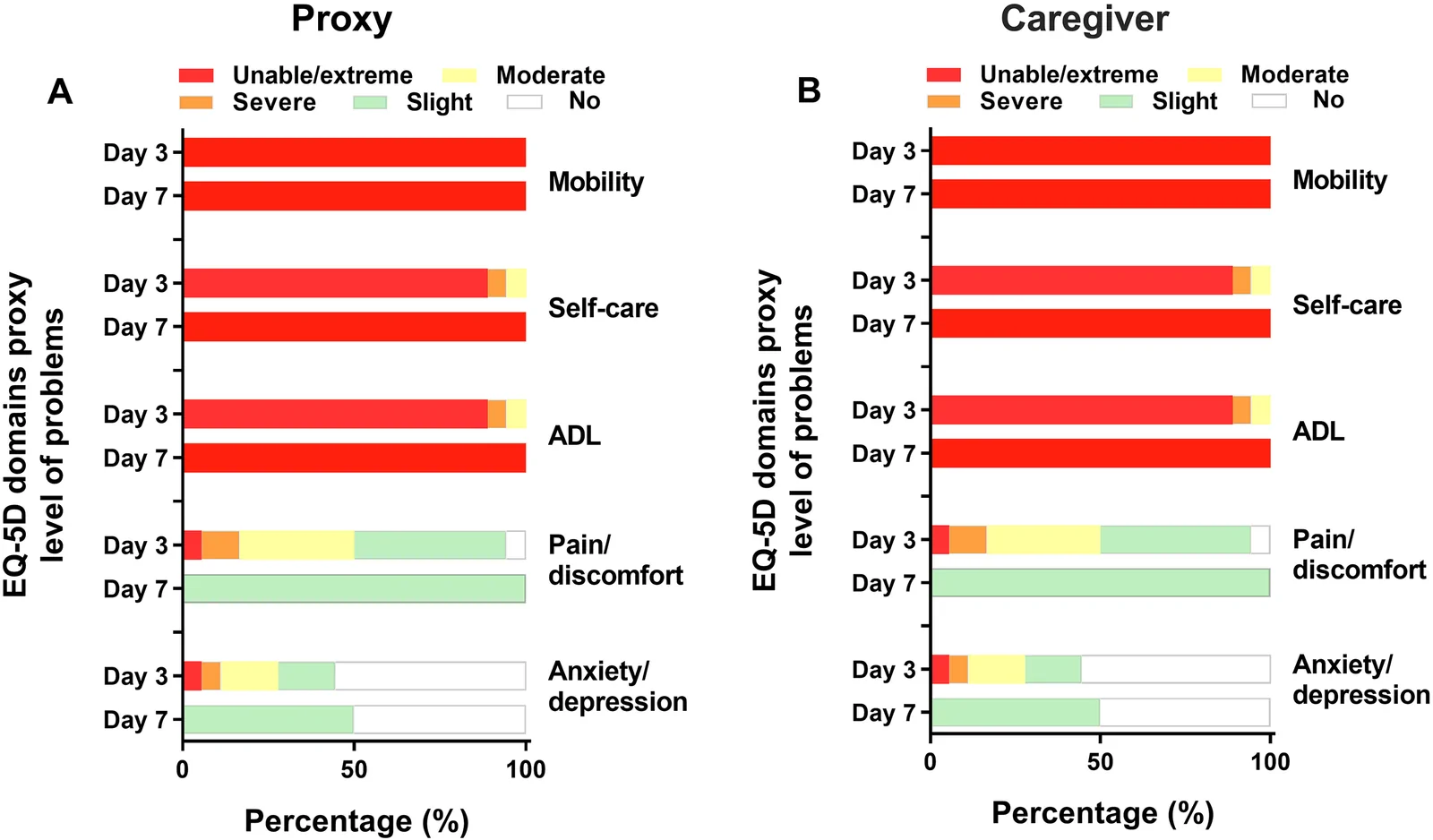
EQ-5D domain scores as rated by proxies and caregivers on day 3 and 7. Data are presented as percentage of patients still alive at the time of the analysis.

The median EQ-5D scores at day 3 were low (median 0.30) as ranked by de proxy and caregivers (Table 2). All patients were unable to mobilize, had a high degree of ADL dependency, and were limited in daily life activities (Figure 1). There was a low degree of neuropsychiatric symptoms with a low impact on caregivers according to the NPI-Q (Table 2). The Qualidem scores are also displayed in Table 2.

EQ-5D, EuroQoL-5D-5L.

**Table 2.** EQ-5D Utility and VAS Scores for Proxies and Caregiver, PACSLAC, NPI-Q Scores and Qualidem Scores.

EQ-5D		N = 20*	Proxy	N = 20*	Caregiver
Utility	Day 3	N = 18	0.30 [0.15-0.32]	N = 19	0.30 [0.03-0.32]
	Day 7	N = 2	0.29 [0.25-]	N = 2	0.14 [-0.04-]
VAS	Day 3	N = 17	25 [15-40]	N = 19	30 [9-50]
	Day 7	N = 2	14 [8-]	N = 2	10 [9-]
PACSLAC (≤4)					
Rest	Day 3	N = 19	13 (68%)		
	Day 7	N = 2	1 (50%)		
Nursing care	Day 3	N = 19	2 (11%)		
	Day 7	N = 2	1 (50%)		
NPI-Q	Day 3		Present (yes)	Total domain score	
Delusions		N = 19	4 (21%)	0 [0-0]	
Agitation/aggression		N = 19	1 (5%)	0 [0-0]	
Depression/Dysphoria		N = 19	0 (0%)	N.A.	
Anxiety		N = 19	6 (32%)	0 [0-2]	
Euphoria		N = 19	1 (6%)	0 [0-0]	
Apathy/Indifference		N = 19	2 (11%)	0 [0-0]	
Disinhibition		N = 19	0 (0%)	N.A.	
Irritability/Lability		N = 19	2 (11%)	0 [0-0]	
Aberrant motor behaviors		N = 19	3 (16%)	0 [0-0]	
Nocturnal restlessness		N = 19	2 (11%)	0 [0-0]	
Change in appetite		N = 19	1 (5%)	0 [0-0]	
Hallucinations		N = 19	3 (16%)	0 [0-0]	
Total score (0-96)		N = 19	4 [0-11]		
Qualidem day 3					
Care relationship (0-21)		N = 15	20 [18-21]		
Positive affect (0-18)		N = 15	10 [6-11]		
Negative affect (0-9)		N = 15	9 [7-9]		
Restless tense behavior (0-9)		N = 15	5 [3-7]		
Positive self-image (0-9)		N = 15	9 [8-9]		
Social relations (0-18)		N = 15	8 [6-10]		
Social isolation (0-9)		N = 15	6 [4-6]		
Feeling at home (0-12)		N = 15	12 [11-12]		
Having something to do (0-6)		N = 15	0 [0-5]		

Data are presented as median (P_25_-P_75_) or as number (%).

EQ-5D, EuroQoL-5D-5L; VAS, visual analog scale; PACSLAC, the Pain Assessment Checklist for Seniors with Limited Ability to Communicate; NPI-Q, Neuropsychiatric Inventory–Questionnaire; N.A., not applicable.

*Discrepancies with the number of included patients and number of completed questionnaires are either to due missing data or patients at the final stage of the dying process at the time of conducting the questionnaires with caregivers or proxies.

At rest, 68% of the patients (n = 13) did not experience pain according to the PACLSAC at day 3. During nursing care, this decreased to 10% (n = 2) (Table 2). The degree of pain was relatively well controlled according to the EQ-5D in most patients as 83% (n = 15) and 74% (n = 14) had “none- to moderate” pain or discomfort according to proxies and caregivers, respectively (figure 1). All patients were administered morphine during follow-up. The daily equivalent administration of 1 mg of oral morphine was 39 mg [16-65], which was mostly administered subcutaneously. During the study period, antipsychotic drugs were administered to two patients (10%) and sedatives to 18 (90%) of the patients. In 15 patients (83%), all medication other than analgesia and sedatives for comfort was discontinued at day 3.

During the first 3 days, 17 patients (85%) were bed-ridden. One patient was able to transfer into a wheelchair and two patients were able to make a few steps under guidance of a physiotherapist. All patients eventually became bed-bound. At day 3, only eight patients (35%) were conscious and able to communicate.

The 7-day mortality rate was 90% with a median time to death of 5 days [3-6]. The 14-day mortality was a 100% (n = 20). In 14 cases (70%), palliative sedation was conducted according to the treating physician. Four adverse events, all Clavien-Dindo grade II, were registered in three different patients (15%). These were two cases of a delirium, one case of a pressure ulcer (Grade II), and one case of clinical suspicion of further fracture dislocation after initial attempts of patient mobilization.

**Figure 2.**
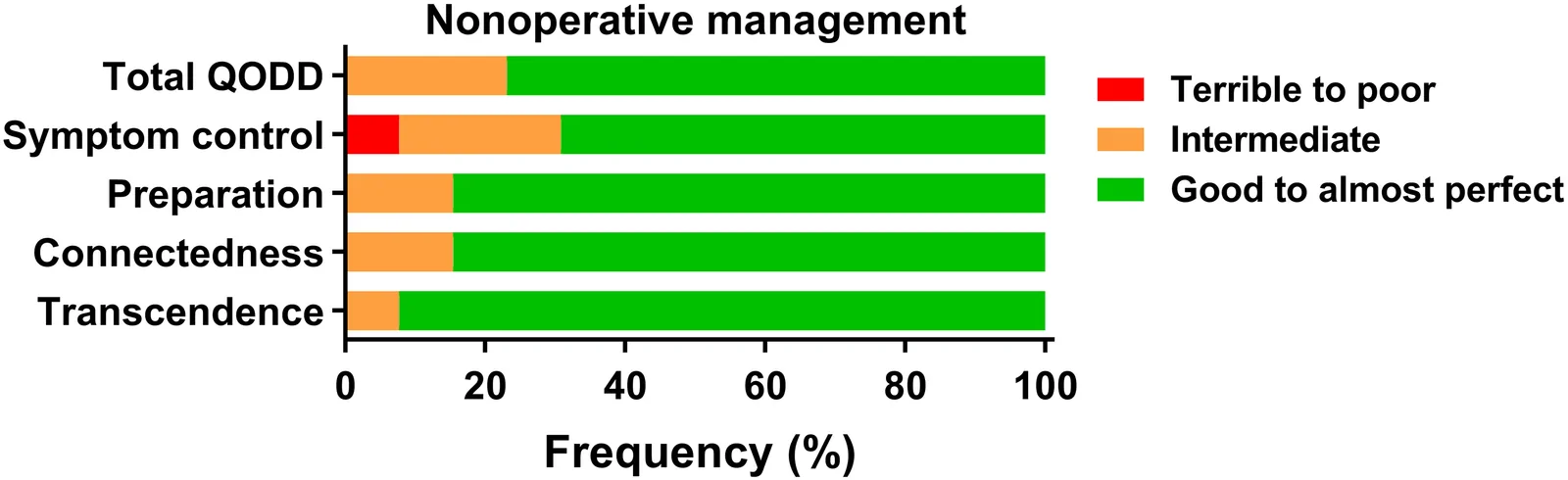
QODD clinical domain scores (N = 13). Frequencies are reported as percentages (%) of total respondents. QODD, quality of dying and death.

**Figure 3.**
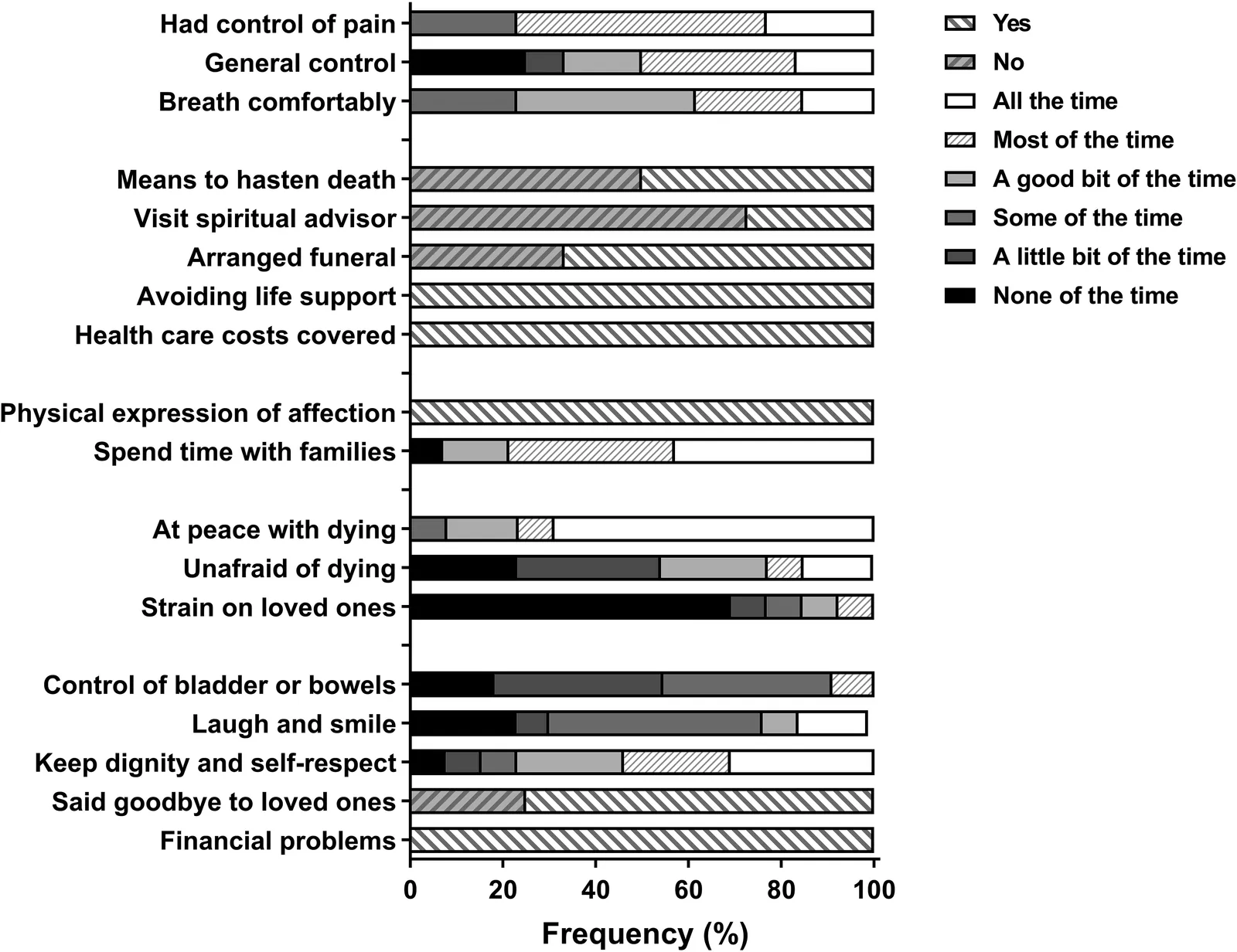
Percentages of if or to what extent items occurred during the final period of the decedent's life.

The overall quality of the dying was good to almost perfect in 77% of the patients (n = 10/13) and intermediate in 23% (n = 3/13) (Figure 2). Symptom control was rated as good to almost perfect in 70% of the cases, intermediate in 23% of the cases, and terrible to poor in one case (8%). An overview of every aspect of the quality of the dying process is shown in Figure 3.

## Discussion

This study showed that NOM of a suspected proximal femoral fracture in nursing home patients with a DNH directive results in high treatment satisfaction. Predictably most patients were bed-ridden with high ADL dependency leading to low HRQoL scores. However, these HRQoL scores are comparable to operatively treated patients in the first week post-surgery in a previous study.^2^ As expected, there was a short life expectancy, but the quality of dying was high while adverse event rates were relatively low with few neuropsychiatric symptoms. It can therefore be seen as a humane treatment option for those frail nursing home patients with a limited life expectancy who sustain a proximal femoral fracture. To our knowledge, this is the first study in international literature that provides an in-depth analysis of the clinical course in palliative nonoperatively managed nursing home patients with a suspected hip fracture and a DNH.

### Goals of Care

It is known that a large proportion of nursing home residents have goals of care that are mainly focused on QoL and comfort.^21,22^ The results of this study show that these goals of care are also achieved with palliative NOM while forgoing hospital admission in case of a suspected proximal femoral fracture. DNH directives potentially spares patients from unnecessary, painful transfers, diagnostic procedures without clinical consequences, the risks associated with hospital admission, and ultimately reduces health care costs without altering the quality of care as proven with the high treatment satisfaction, low adverse events rate, and high quality of dying in this cohort. Previous studies have shown that forgoing hospital transfer for a potential hip fracture could result in an average costs reduction of €2226 (SD 925).^23^ However, the costs of a surgical procedure or hospital admission should not influence treatment making decisions.^23^

### Advance Care Planning

The outcomes of this study stress the importance of ACP as there is potentially no additional value of hospitalizing patients with a suspected hip fracture that will not be treated surgically due to their frailty or immobility. This is important to take in to consideration, as previous research has suggested that almost 20% of nursing home residents are hospitalized in the final month of life of which hospital admission with trauma or injury being the second most common cause for these admissions.^24^ Advance are planning potentially prevents these unnecessary hospital admissions without clinical consequences. ACP is known to positively impact the quality of end-of-life care and improves compliance with patients’ end-of-life wishes and patients’ and their families’ satisfaction with care. Moreover, it reduces family stress, anxiety, and depression.^25^ Early integration of palliative care with a DNH directive in case of a proximal femoral fracture therefore can only contribute to the QoL and treatment satisfaction, and death in these patients should not be seen as preventable or as a failure of treatment.

It must be realized that unlike chronic conditions with a longer disease course such as dementia or other degenerative neurologic disorders, a proximal femoral fracture is an acute event that requires prompt decision making. Therefore, these patients are at risk for hospitalization in case DNH directives are not in place. This also stresses the importance of ACP. Currently approximately 20% of nursing home residents have a DNH directive.^22,26^ While a hip fracture is an acute event, nightly emergency department admission via on-call physicians who are not familiar with the patients history are not expedient. The decision to potentially forgo hospital admission could also be extended to the next day in case their usual treating physicians will then be present without altering clinical outcomes in order to prevent unnecessary hospital admission.

### Indications for Do-Not-Hospitalize Directives

However, NOM may not be the best option for every nursing home patient, and the indications greatly depend on the degree of frailty. Proper patients selection for NOM is pivotal as inadequate patient selection might also result in adverse outcomes.^2^ Therefore, if there are doubts about the best treatment decision, hospitalization with pelvic radiographs to ensure the presence of the fracture followed by a process of transmural shared decision making seems te best way forward to ensure the best treatment decision.

### Nonoperative Management

In case of palliative NOM for suspected hip fractures, the focus should be on comfort, symptom management, and QoL. This often entails adequate pain management with morphine and/or sedatives with few attempts of mobilization as this is often painful and unsuccessful and potentially results in further fracture dislocation. While it is known that symptom control is the most challenging aspect in NOM of proximal femoral fractures, this was generally well controlled in this cohort of patients without any peripheral nerve blocks.^2,27^

### Limitations

This study does have some limitations. The sample size limits generalizability and could be subject to selection bias. It was unknown how many suspected proximal femoral fractures occurred during the study period and how many were missed and/or hospitalized so no estimations could be made of the prevalence of the NOM with a DNH directive or degree of selection bias. The response of proxies on the QODD questionnaire was relatively low (65%) and could result in bias; however, results are consistent with a previous study on NOM.^2^

Overall, it can be concluded that NOM of suspected proximal femoral fractures leads to desired clinical outcomes with high treatment satisfaction, high quality of dying, good symptom control, and few adverse events. These outcomes will aid further decision making, ACP, the anticipation of death, and provide realistic expectation management. The decision to refrain from medical treatment and/or NHD may also apply to other medical emergencies, for example cerebrovascular accidents or severe respiratory infections. Further research is needed to determine whether the conclusion of this study can be extended to other treatment dilemmas in the nursing home population.

## Conclusions

This study showed that NOM of a suspected proximal femoral fracture in nursing home residents with a DNH directive, results in high treatment satisfaction, high quality of dying with good symptom control, and predictable short-term mortality rates.
